# Synthesis of highly transparent ultrananocrystalline diamond films from a low-pressure, low-temperature focused microwave plasma jet

**DOI:** 10.1186/1556-276X-7-82

**Published:** 2012-01-19

**Authors:** Wen-Hsiang Liao, Da-Hua Wei, Chii-Ruey Lin

**Affiliations:** 1 Department of Mechanical Engineering and Institute of Manufacturing Technology, National Taipei University of Technology, Taipei, 106, Taiwan; 2 Graduate Institute of Mechanical and Electrical Engineering, National Taipei University of Technology, Taipei 106, Taiwan

**Keywords:** ultrananocrystalline diamond films, focused microwave plasma jet, low-pressure/low-temperature synthesis, transmittance

## Abstract

This paper describes a new low-temperature process underlying the synthesis of highly transparent ultrananocrystalline diamond [UNCD] films by low-pressure and unheated microwave plasma jet-enhanced chemical vapor deposition with Ar-1%CH_4_-10%H_2 _gas chemistry. The unique low-pressure/low-temperature [LPLT] plasma jet-enhanced growth even with added H_2 _and unheated substrates yields UNCD films similar to those prepared by plasma-enhanced growth without addition of H_2 _and heating procedure. This is due to the focused plasma jet which effectively compensated for the sluggish kinetics associated with LPLT growth. The effects of pressure on UNCD film synthesis from the microwave plasma jet were systematically investigated. The results indicated that the substrate temperature, grain size, surface roughness, and *sp^3 ^*carbon content in the films decreased with decreasing pressure. The reason is due to the great reduction of *H*_α _emission to lower the etching of *sp^2 ^*carbon phase, resulting from the increase of mean free path with decreasing pressure. We have demonstrated that the transition from nanocrystalline (80 nm) to ultrananocrystalline (3 to 5 nm) diamond films grown via microwave Ar-1%CH_4_-10%H_2 _plasma jets could be controlled by changing the pressure from 100 to 30 Torr. The 250-nm-thick UNCD film was synthesized on glass substrates (glass transition temperature [*T*_g_] 557°C) using the unique LPLT (30 Torr/460°C) microwave plasma jet, which produced UNCD films with a high *sp^3 ^*carbon content (95.65%) and offered high optical transmittance (approximately 86% at 700 nm).

## Introduction

The ultrananocrystalline diamond [UNCD] films are outstanding material candidates for multifunctional device applications and attracting strong scientific and technological interests due to their unique properties stemming from their ultrafine (< 10 nm) grains and a pure diamond phase, such as high wear resistance [1], optical transparency from deep UV to far infrared [2,3], chemical stability, excellent electron field emission [4,5], and superior capacity to incorporate n-type dopants in addition to a smooth surface [6-9]. However, improving the syntheses and applications of UNCD films for functional devices and components highly requires the development of a new low-temperature and low-pressure process for wider uses in substrates and an effective growth with low consumption of source gases, besides optimizing the performance of UNCD films by controlling the pre-growth seeding and growth parameters [10].

Microwave plasma chemical vapor deposition [MPCVD] from Ar-1%CH_4 _gas chemistry was typically used to synthesize UNCD films in order to greatly enhance plasma species activity and diamond secondary nucleation [10-12]. The normal growth temperature and pressure of UNCD films synthesized by microwave Ar-1%CH_4 _plasma without addition of H_2 _were 800°C and above 100 Torr, respectively [10-12]. The growth temperature depended on the substrate and plasma heating during synthesis. Commonly, the plasma heating cannot be avoided during UNCD growth; thus, minimizing the plasma heating is crucial to realize a low-temperature synthesis. Therefore, argon-rich (hydrogen-poor) microwave plasma is popularly adopted for low-temperature preparation of UNCD films due to the much lower thermal conductivity of argon and much less power levels required for argon plasma formation compared with hydrogen [10].

The microwave plasma jet-enhanced chemical vapor deposition [MPJCVD] for UNCD film synthesis developed in our lab takes several advantages compared with the regular MPCVD process, namely which can improve the density and activity of plasma species through excitation of the focused plasma jet [13,14], enabling it to achieve high-efficiency and high-quality deposition at low-pressure/low-temperature [LPLT] conditions (without substrate heating). The MPJCVD-enhanced growth [MEG] is particularly critical in LPLT deposition to compensate for the insufficient density and kinetics of growth species associated with LPLT synthesis. Therefore, we describe here a unique plasma jet technique to successfully grow UNCD films at LPLT (30 Torr/460°C) that yields films with smooth surface, pure diamond nanograins (3 to 8 nm), and high optical transmittance in the visible light region using relatively low pressure, low power (700 W), and even with addition of H_2 _(Ar-1%CH_4_-10%H_2_) compared with the typical plasma processes [10-12]. The highly transparent UNCD films were grown directly on glass substrates with a low glass transition temperature (*T*_g _557°C). The process opens further feasibility for the LPLT synthesis of UNCD films, providing a promising platform fabrication for diamond-based multifunctional devices and coating on low-melting point materials with low cost. The synthesis and characteristics of UNCD films produced by the MEG technique at various growth pressures (5 to 100 Torr) and temperatures (400°C to 700°C) were systematically studied by *in situ *optical emission spectroscopy [OES], visible Raman spectroscopy, synchrotron-based X-ray absorption near-edge structure [XANES] spectroscopy, atomic force microscopy [AFM], field-emission scanning electron microscopy [FESEM], and field-emission transmission electron microscopy [FETEM].

## Experimental details

The diamond films were synthesized using the homemade MPJCVD system. The plasma jet was induced in Ar-1%CH_4_-10%H_2 _gas chemistry at a microwave power of 700 W. The total pressure of reactant gas was varied from 5 to 100 Torr (5, 15, 30, 60, 80, and 100 Torr) in the synthesis of diamond films. The deposition process was carried out without heating the substrates. The substrate temperature was influenced only by plasma jet heating at various pressures from 5 to 100 Torr, which increased from approximately 400°C to 700°C with increasing pressure. The growth rate of the diamond films using plasma jet was gradually increased with increasing pressure, approximately 0.25 μm/h at 30 Torr and approximately 0.97 μm/h at 100 Torr. The thickness of the diamond films was confirmed by a FESEM image of the cross section. N-type Si wafers with a (100) orientation were initially used as substrates for the deposition of diamond films at various pressures. The glass substrates with *T*_g _of 557°C were applied to support LPLT UNCD films for the fabrication of highly transparent coatings and further confirmed the successful synthesis of UNCD films at a LPLT condition without any damage to the substrates. Pretreatment on the substrates was performed by the spin coating of a diamond nanoparticle solution to enhance nucleation at low temperature [13].

The as-grown films were characterized by FESEM (S-4800, Hitachi, Chiyoda-ku, Tokyo, Japan), visible Raman spectroscopy (micro-Raman, Renishaw Inc, Taichung, Taiwan), synchrotron-based XANES spectroscopy (Carbon K-edge spectra with a resolution of 0.1 eV, total electron yield mold, at the Dragon BL11A beamline of the National Synchrotron Radiation Research Center in Taiwan), FETEM (Tecnai F30, Philips, Best, The Netherlands), and AFM (NS3a, Digital Instruments, Santa Barbara, CA, USA) for obtaining comprehensive information on the surface morphology, roughness, atomic bonding nature, and detailed nanostructural characterizations. Optical transmission spectrum of the as-grown UNCD films ranging from 350 to 950 nm was characterized with a UV-A/Visible/near-IR spectrophotometer (MP100-M, Mission Peak Optics, Fremont, CA, USA). The focused microwave plasma jet was analyzed during synthesis by *in situ *OES (BTC112E, B&W TEK, Newark, DE, USA) to explore the species composition at different growth processes.

## Results and discussion

**Figure 1:**
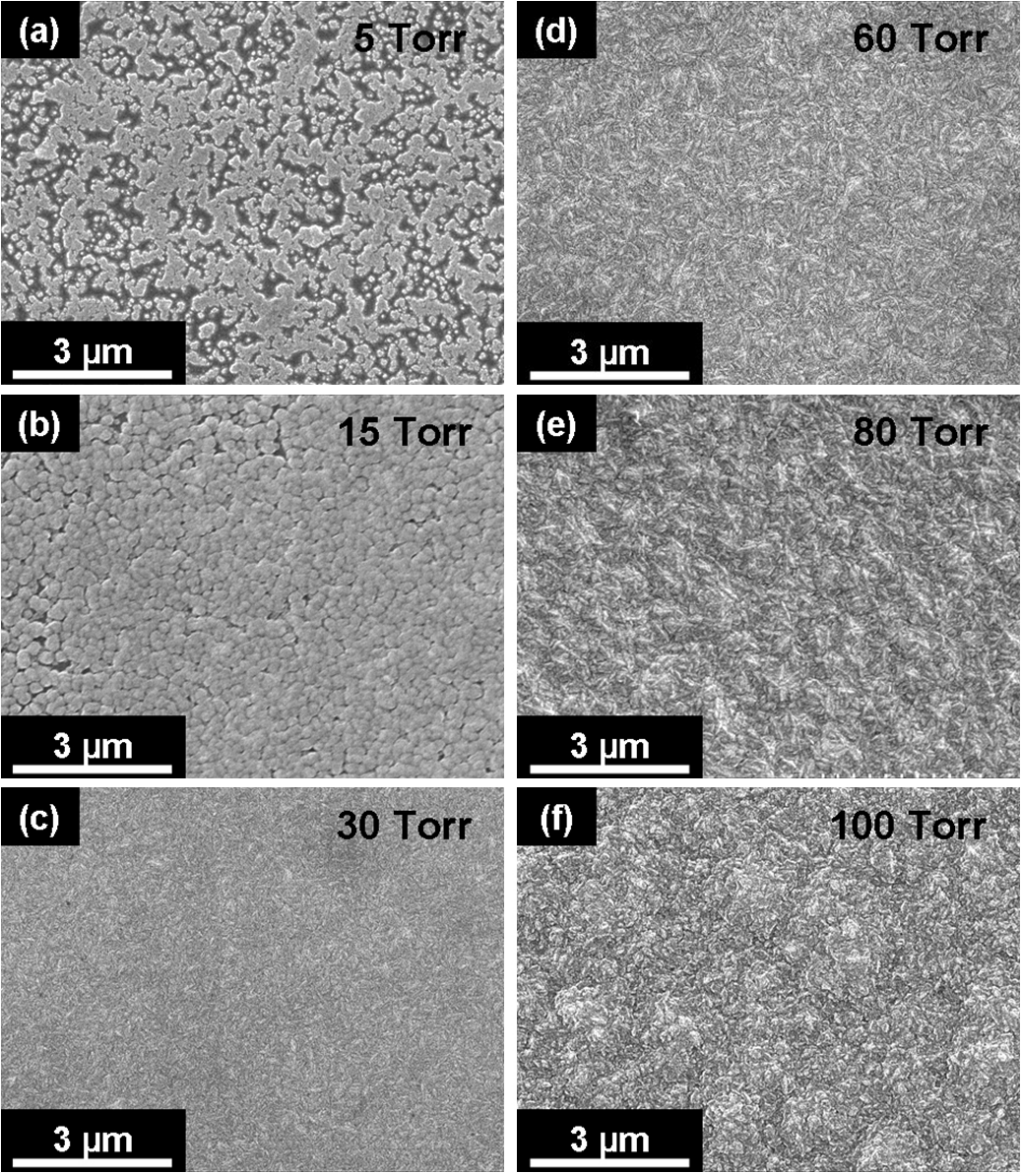
**SEM images of the diamond films grown by microwave Ar-1%CH_4_-10%H_2 _plasma jet at various pressures**. (**a**) 5, (**b**) 15, (**c**) 30, (**d**) 60, (**e**) 80, and (**f**) 100 Torr.

Plan-view SEM micrographs shown in Figure 1 demonstrated an obvious change in the surface morphology of as-grown films while pressures are increased from 5 to 100 Torr in the MEG process. The apparently discontinuous film shown in Figure 1a indicated that the least effective deposition was at the pressure of 5 Torr. The film deposited at 15 Torr still has few remaining vacant sites but almost fully covered the Si substrate as shown in Figure 1b. For the deposition at a pressure of 30 Torr (Figure 1c), a uniform and smooth film composed of very fine grains was obtained without any visible pinholes. This condition is employed to estimate a minimum demand for growth pressure to obtain a dense and continuous film from the focused microwave plasma jet. A further increase in pressure induced the diamond film's surface to form an elongated cluster with a needle-like structure of about 300 nm in length, as shown in Figure 1d, e. Figure 1f shows that the film grown at 100 Torr would form distinctly greater clusters and a rougher surface morphology compared to nearly invisible boundaries at a growth pressure of 30 Torr (Figure 1c). With the increase in growth pressure, the grain size of the diamond films seems to gradually increase with increasing cluster size and surface roughness. However, the exact size of the nanocrystallites cannot be clearly identified by SEM due to the limited resolution, and the details were further explored and discussed below.

**Figure 2:**
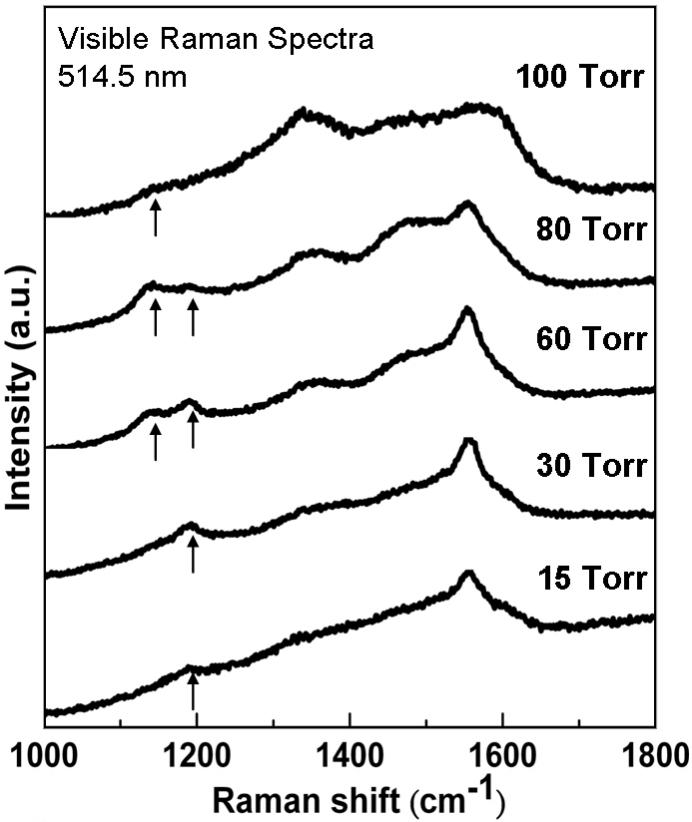
**Visible Raman spectra of diamond films grown by microwave Ar-1%CH_4_-10%H_2 _plasma jet at various pressures**.

Figure 2 shows the visible (wavelength 514.5 nm) Raman spectra of the diamond films grown by the MEG process at various pressures from 15 to 100 Torr. Raman spectra of as-grown films typically reveal nanocrystalline diamond [NCD] features [10,15]. The peak of the *sp^3^*-bonded carbon (diamond) around 1, 332 cm^-1 ^has disappeared or is overlapped by the D (disordered) band of the *sp^2^*-bonded carbon (non-diamond) around 1, 350 cm^-1 ^while the films were grown at 15, 30, and 60 Torr. The reason is due to the diamond films consisted of nanocrystallites with a higher proportion of grain boundaries [GBs] which enhanced the much higher sensitivity of *sp^2 ^*bonding over *sp^3 ^*bonding by visible Raman [10,16,17]. The sharp peak intensity of the *sp^3^*-bonded carbon is increased in spectra of the films grown at relatively high pressure (80 and 100 Torr), indicating that the quality of the diamond films was gradually improved as the pressure increased. Simultaneously, the decrease and broadening of the G (graphitic) band at 1, 560 cm^-1 ^with increasing pressure demonstrate the *sp^2 ^*fraction reduction in the films, also implying that the grain size was increased due to the decrease of the GBs proportion [14,18]. This is in accord with the observation in the SEM images (Figure 1).

Two trans-polyacetylene [t-PA] bands were observed in the spectra at approximately 1, 140 and 1, 480 cm^-1 ^while the films were grown at above 60 Torr, which represented the NCD structures that existed at the GBs in the films [15,19,20]. Interestingly, the spectra were found to show a peak centered at approximately 1, 190 cm^-1 ^as the synthesis was at a pressure from 15 to 80 Torr. The decrease in this peak with increasing pressure is opposite to the t-PA bands in the spectra. This phenomenon likely originated from the difference in the crystal size between UNCD and NCD films. Moreover, the concurrent absence or weakening of the peaks located at 1, 190 and 1, 140 cm^-1 ^(t-PA) for the sample grown at 100 Torr suggested that the content of C-H bonds (t-PA) in the films would be decreased with increasing pressure (5 to 100 Torr) and temperature (400°C to 700°C). A decrease in the relative GB fraction for t-PA bands existed, and the increase in the substrate temperature was to promote hydrogen desorption from the films [10,18]. The decrease of hydrogen trapping during synthesis is expectably caused by high-temperature growth (high-pressure) due to the hydrogen desorption temperature which is between 600°C and 1, 000°C [21]. The above features of visible Raman spectra are similar to those of the UNCD films deposited by microwave Ar-1%CH_4 _plasma at various substrate heating [10], but the bonding structure and quality of the films are controlled by pressure via the MEG process, suggesting that the MEG process could improve the synthesis of the UNCD films at LPLT without substrate heating.

**Figure 3:**
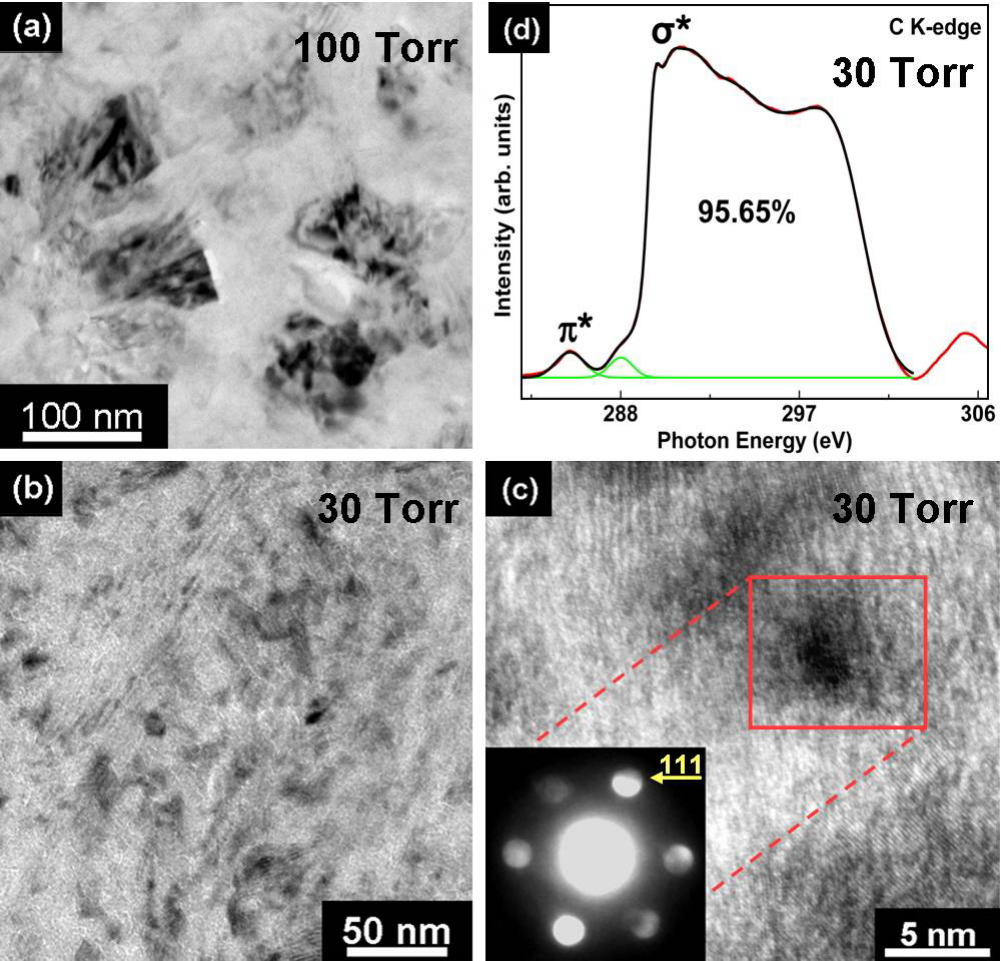
**TEM images and XANES spectrum**. Plan-view TEM image of the diamond film grown by the microwave Ar-1%CH_4_-10%H_2 _plasma jet at (**a**) 100 and (**b**) 30 Torr. (**c**) Enlarged TEM image of (b); the inset shows the corresponding NBD pattern of a single diamond nanograin. (**d**) The XANES spectrum of the UNCD film grown from the LPLT (30 Torr/460°C) plasma jet technique.

Figure 3a shows the plan-view TEM image of the diamond film grown at 100 Torr, which reveals distinctly that the grain size is approximately 80 nm with a roundish geometry. The plan-view TEM image shown in Figure 3b illustrated that the diamond film grown at 30 Torr consisted of ultrananosized (3 to 8 nm) crystallites (UNCD) uniformly dispersed in an amorphous carbon matrix. Figure 3c shows the enlarged TEM image of the MEG UNCD films grown at 30 Torr, and the inset shows the corresponding nanobeam diffraction [NBD] pattern of a single diamond nanograin (approximately 5 nm) with a spherical shape. The beam diameter used for NBD was approximately 15 nm, allowing for the diffraction pattern from only one or a few diamond nanograins to be observed. The diffraction pattern shows discs of intensity for the {111} planes of diamond, indicating that a single nanograin is a single crystalline diamond [22]. To definitely distinguish between the *sp^2 ^*and *sp^3 ^*bonds in hybridized carbon materials, C K-edge XANES spectrum has been applied as shown in Figure 3d. Figure 3d clearly indicates that a typical fine structure for MEG UNCD films (30 Torr/460°C) is a cubic diamond (C 1*s *core exciton at approximately 289.7 eV and C-C 1*s *→ σ* hybrid bonds between approximately 290 and 302 eV) of 95.65% with a small fraction of the *sp^2^*-bonded carbon (C = C 1*s *→ π* at approximately 285.3 eV) and C-H bonding (C-H 1*s *→ σ* at approximately 287.5 eV) at GBs [23,24]. The TEM analyses confirmed that the grain size of the diamond films decreased (from 80 nm to 3 to 8 nm) with decreasing pressure (100 to 30 Torr) and consisted with the previous SEM (Figure 1) and Raman analyses (Figure 2). The TEM and XANES analyses also further confirmed that UNCD films could be successfully synthesized at LPLT by a microwave Ar-1%CH_4_-10%H_2 _plasma jet without substrate heating, which is identical to grain size distribution and atomic bonding characteristics of the UNCD films grown by a microwave Ar-1%CH_4 _plasma with substrate heating.

**Figure 4:**
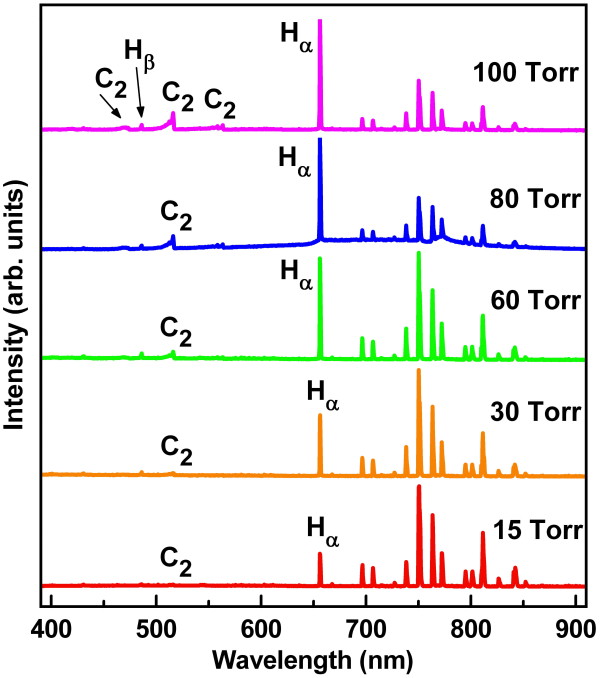
***In-situ *OES spectra of diamond films grown by microwave Ar-1%CH_4_-10%H_2 _plasma jet at various pressures**.

**Figure 5:**
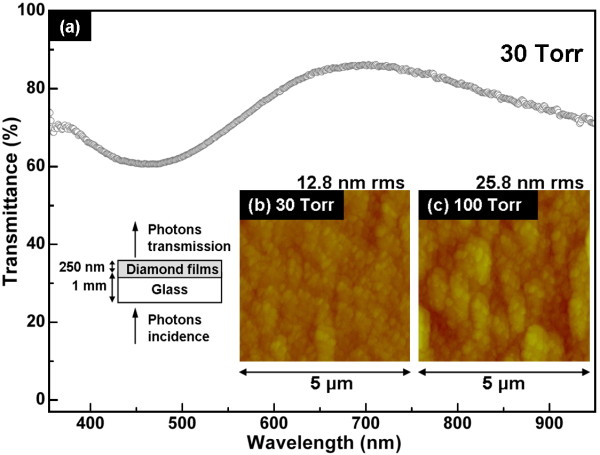
**Optical transmittance spectrum and AFM images**. (**a**) Optical transmittance spectrum of the UNCD films grown by the LPLT (30 Torr/460°C) MEG process with a thickness of approximately 250 nm. (**b**) The corresponding AFM image of MEG UNCD films. (**c**) AFM image of diamond films grown at 100 Torr.

The *in situ *OES spectra (Figure 4) were performed to diagnose the species composition in the Ar-1%CH_4_-10%H_2 _plasma jets in order to understand the growth behavior resulting from the increase in growth pressure and temperature to lead to such changes on structural and bonding characteristics of diamond films. OES spectra reveal that an excited intensity of *H*_α _(656.2 nm) species is increased markedly with increasing pressure and dominant in the plasma jets with a pressure over 80 Torr. However, the decrease in Ar emissions (over 700 nm) with increasing pressure is contrary to the *H*_α _emission in the spectra. The high hydrogen atom concentration during synthesis could promote the etching of *sp^2 ^*carbon phase and reduce the diamond renucleation [25]. Thus, the grain size and *sp^3 ^*bonds in the diamond films would be increased at relatively high pressure, resulting in the NCD films (80 nm) synthesized at 100 Torr with a rougher surface (Figure 5c). Moreover, the increase in the mean free path of plasma species with decreasing pressure led to a greatly decreased amount of atomic hydrogen emission (hydrogen-poor) during synthesis and evidently, the creation of a low-temperature environment for UNCD films growth from low-pressure MEG process with addition of H_2 _[10].

The transmittance spectrum of the UNCD film with a thickness around 250 nm grown from the LPLT MEG process on the glass substrate was measured in the range of 350 to 950 nm (Figure 5a). The scheme of the measurement is illustrated in the bottom left inset of Figure 5. The optical transmittance of the as-grown UNCD film is oscillated due to the interference effect during the photon transmission in the film, resulting in the variation of transmittance from 60% at 450 nm to 86% at 700 nm. The transmittance of the diamond films is dominated by the surface smoothness and diamond (*sp^3 ^*bonding) content in the films [26]. The film grown at 30 Torr/460°C consisted of pure diamond nanocrystallites (3 to 8 nm) without any thermal damage to the substrate, which retained a high degree of diamond purity (95.65%) but revealed a far smoother surface (12.8 nm root-mean-square [rms]) to minimize light scattering from the surface of the diamond films (Figure 5b), resulting in the outstanding optical transparency obtained from the LPLT synthesis. The transmission analysis complemented the SEM, TEM, AFM, XANES, visible Raman, and OES analyses to constitute convincing evidence of successful fabrication of highly transparent UNCD films at LPLT condition and completed investigation of the relationships between the growth conditions, nanostructures, and material properties of the diamond films synthesized from the focused microwave Ar-1%CH_4_-10%H_2 _plasma jet at different growth processes.

## Conclusions

A no-heating LPLT MEG technique has been developed successfully to synthesize UNCD films on glass substrates with high transparency (approximately 86% at 700 nm), smooth surface (approximately 12.8 nm rms), uniform diamond nanocrystallites (3 to 8 nm), and very high *sp^3 ^*content (95.65%) using relatively low-output power (700 W), low Ar gas chemistry (Ar-1%CH_4_-10%H_2_), low pressure (30 Torr), and even low temperature (460°C) compared with the typical microwave Ar-1%CH_4 _plasma with heating procedures. The synthesis of UNCD films using the uniquely focused plasma jet was confirmed to efficiently compensate for the sluggish kinetics and insufficient density of plasma species during the LPLT synthesis. A new and effective way to control the crystal size, surface morphology, and growth mechanism of diamond films by regulating the growth pressure in a systematic study was reported. Based on the TEM images of all films, it has been demonstrated that the transition of grain size from NCD (80 nm) to UNCD (3 to 8 nm) films controlled by the pressure ranged from 100 to 30 Torr. The reason is due to the increase of the mean free path for the excitation of plasma with decreasing pressure, resulting in a decreased amount of atomic hydrogen emission to greatly lower the etching of the *sp^2 ^*carbon phase during synthesis. The NBD and XANES characterizations further demonstrated the ultrananocrystalline diamond nature of the films grown from the focused microwave Ar-1%CH_4_-10%H_2 _plasma jet at LPLT condition.

## Competing interests

The authors declare that they have no competing interests.

## Authors' contributions

W-HL and D-HW conceived and designed the experiments, analyzed the results, and contributed to the writing of the manuscript. C-RL, together with the other authors, revised and approved the final manuscript.
